# Pyorrhea and Prophylaxis

**Published:** 1912-04-15

**Authors:** F. H. Skinner

**Affiliations:** Chicago, Ills.


### PYORRHEA AND PROPHYLAXIS.

BY F. H. SKINNER, D.D.S.,CHICAGO, ILLS.

( Read before the Dental Society of the State of New York, at its annual meeting, Albany, May 4, 1911.)

So much has been written on the subject of oral hygiene, prophylaxis, etc., that it seems as if there were little more to be said thereon. It is not the writer's aim to encompass the whole subject of oral hygiene, which would comprise cleanliness of the nasal passage and sinuses, tonsils, throat, as well as the correction of all irregular and pathological conditions of the mouth and teeth. It is well within our province, however, to be able to diagnose by a glance at one of our younger patients whether he is suffering from adenoids, diseased tonsils, etc., and to refer him to an oral surgeon for the correction of these conditions.

Our field covers the teeth and the mouth, and the object of this paper is to suggest the necessity of impressing upon our patients the need of absolute cleanliness and the methods of maintaining it. This is especially important with our younger patients, as well as with those who have them in their charge.

The following suggestions have proved to be very successful with those practitioners who have given them a thorough trial. Very few are original; they represent rather a medley of ideas applied in my own practice, and gained from reading and discussing with others working in the same field.

### THE INITIAL CAUSES OF CARIES AND PYORRHEA ALVEOLARIS

The exact processes of pyorrhea or of caries have never been fully explained so that the detailed results of one practitioners work are not disputed by another standing equally high in the profession, but it is probably universally agreed that neither pyorrhea nor caries is found if the tooth surfaces have always been kept clean; that all caries is the result of by-products of bacteria thriving in particles of food that have become lodged on the teeth in places where nature has left a defect in development, or on surfaces favorable to the lodgement of food where attrition and the combined action of the tongue, the cheeks, and the lips do not remove it; and lastly, that the debris so lodged is acted upon by acid-producing bacteria. One of the first processes to take place is the formation of gelatinoid plaques or scales, which are so arranged as to bind a destructive mass in proximity to the surfaces of the teeth so that it is removed only by friction, and if it is of long standing, or is attached to a surface which has already become etched from previous attacks, it requires some thing more than the friction of a tooth-brush to remove it.

These deposits usually gather just gingivally to the contact point, around the cervical margins, or in the fissure or in depressions of malformed teeth. If sulfocyanate of potassium, or possibly other allied salts, be present in the saliva these gelatinoid plaques do not form, and such a fortunate individual is immune to caries until a change occurs in the oral fluid.

If, as the debris gathers,there is a sufficient quantity of calcium salt to neutralize any quantity of acid formed, no caries will set in, but a crust will gradually form around the necks of the teeth, and increase in the direction of the root, which if not removed, will finally produce pyorrhea. It is generally observed that when calcium salts gather in abundance no caries is present, and when caries progresses rapidly, calcium salts are absent. The rougher the surfaces of the teeth, the more difficult it is to remove any foreign substance.

It is the essayist's belief that the foundation for a great deal of dental trouble is laid in childhood. During the period of growth it is difficult to induce children to take proper care of their teeth; soft accumulations are left to ferment, and if cavities are not actually formed, the victim enters maturity with the cervical enamel margins roughened and etched. The foundation for trouble is laid before the patient begins to have personal pride enough to care for appearances, or to realize the value of a good set of teeth. About this time the calcium salts which have been used to build up bone tissue are not needed in such large quantity by the system, and are thrown out in the process of elimination. The saliva absorbs its share of these salts, and as it comes in contact with free ammonia, which is always present in the alimentary canal especially if faulty metabolism or intestinal indegestion be present—the calcium salts precipitate and lodge on the nearest hard substance with which they come in contact, usually the teeth, this process going on continually. The catastrophe of dentistry is that a great deal of harm is usually done before patients realize that there is even any danger.

### PYORRHEA ALVEOLARIS

A few weeks ago I asked a specialist in stomach and intestinal indigestion what percentage of troubles in his specialty were caused by pyorrhea and what number of his patients were suffering therefrom. He said if I would reverse the first part of my question he would say that about 95 per cent. of pyorrhea is caused by faulty metabolism and toxemia, and that fully 80 per cent. of his patients had pyorrhea. I differ with him, although recent experiments by Dr. Black seemed to strengthen his argument considerably. Dr. Black claims that by improper or excessive eating he is able to produce calcium salt formation in from six to twelve hours, or that he can prevent it entirely and for an indefinite length of time by proper mastication and diet. In your essayist's opinion, two conditions must always be present to cause pyorrhea. First, the tissue surrounding the teeth must be susceptible to a breaking-down or suppurative process; secondly, there must be a local irritant to abrade the surfaces of the mucous membrane so that infection will take place. In other words, all pyorrhea is of traumatic or infectious origin, and usually both. The following are some of the most common causes: Malocclusion, improper contact points with food impinging upon the gums, ill-fitting bands, any forms of partial dentures which are wholly dependent upon the gums for support or resistance to the opposite occluding teeth, fillings improperly finished at the cervical margins, irregularity, lacerating the tissue with toothpicks in interproximal spaces, a mass of decomposing food, calcium salt deposits, even the minute particles of calculus described by Dr. J. D. Patterson as cactus points, all these will produce inflammation, followed by suppuration, and once suppuration is started, calcium salt deposits develop more rapidly and increase in the direction of the root. Of pyorrhea cases, 75 per cent. are caused, in all probability, by calcium salt deposit which have not been thoroughly removed from under the free margin of the gums when the teeth were being "cleaned," so called.

### DRY PYORRHEA.

Too severe brushing or cemental caries will cause death of the pericementum, the latter most likely by way of infection. Pyorrhea as produced by such conditions is the only form of this disease in which no calcium salt deposit is found.

My time is too limited to elaborate upon the many deteriorating effects of this unwholesome disease on the human system, but I will give just one illustration from my own practice. Two years ago I was called to a house, with instructions to be extremely careful in my procedures and to do only what was absolutely necessary, as the patient had been in bed about three months, and was in an extremely nervous and weakened condition. Upon looking into her mouth, I immediately saw pus exuding from the gums around the necks of all the teeth. I called the nurse's attention to this, and showed her how by gentle pressure on the gum a drop of pus could be pressed out from the tissue around any tooth I selected. I inserted two cement fillings, which required about thirty minutes, then again pressed the gums around the teeth, and was able to produce another drop of pus from every one. The nurse called me aside and asked if I didn't think that this oral condition had something to do with the intestinal infection, which seemed to be the only cause preventing the patient's recovery, and for which the attending physician had been unable to account. He seemed to think that if this intestinal infection could be remedied, the patient would gain at once. The nurse told him of the condition of the teeth and gums. The next day I received a message to be as careful as possible, but to arrest the flow of pus. It required several sittings to go over all the teeth. In the meantime the patient used a mouth-wash every thirty minutes so as to prevent the flow of pus into the stomach as much as possible. Three days after I had finished treatment, the patient was able to take dinner with her family, and subsequently her improvement was steady and rapid.

I do not know just to what extent the amount of pus swallowed was responsible for this patient's illness, but if the tissues around each one of the thirty teeth present exuded one drop of pus every thirty minutes, the patient in twentyfour hours would have swallowed three ounces of pus. Even if only one and one-half ounces of pus were swallowed every day conclusions can readily be deduced as to the effects on an already debilitated constitution.

### TREATMENT.

So far, no known systemic treatment has been efficient in arresting this suppurative process, but the complete removal of the local irritant followed by prophylactic treatment invariably produces satisfactory results.

Any set of instruments with which all deposits, as well as dead pericementum, can be removed, leaving a smooth surface, can be used in curing pyorrhea, providing there is no bone complication or absorption of the root and all other irritants are removed. (I have never seen a tooth which was extracted on account of a pyorrheal condition which did did not show upon minute and careful examination some local cause for the continued irritation.) The set designed by C. M. Carr has proved the most satisfactory in the essayist's hands. This set of instruments is designed so that every portion of any normally shaped tooth can be reached and planed, with the exception of the sharp angle formed by bifurcating roots, where an instrument of the Younger type is indicated. The point of the Carr instrument is so shaped that accurate results are obtained, with less pain to the patient than was caused by the instruments heretofore used. Should the tissues be hypersensitive, a drop of "Acestoria" acts as an anesthetic as well as a mild antiseptic. Of course, if the pockets are deep, they should be kept clean until new periosteum and cancellous bone have formed—if the pockets show a tendency to fill— or until the gums have receded down to a good attachment. This may require from a few days to a year or so. The tissues frequently are benefited by by stimulating with lactic acid, the iodin preparations, phenol-sulfonic acid, bifluorid of ammonia—Head's tartar solvent—or zinc chlorid, etc., but getting the teeth clean and smooth and maintaining cleanliness is the procedure most necessary to cure this curse of the human race. After the tooth has been thoroughly scaled, smoothed, and polished, far more important than medication is the following up, at successive sittings, of the polishing of the tooth to the very bottom of the pocket. If a pyorrhea pocket does not heal, it is because the tooth had not been thoroughly planned or scaled, smoothed, and polished, or cleanliness has not been maintained, and a reinfection has taken place.

Attention should be called again to the benefit derived from polishing the tooth surface clear to the botton of the pocket, if possible, at the first sitting as well as at subsequent treatments, which should be administered every three or four days until the pocked is entirely healed. A very fine wood point, such as a toothpick, held in a suitably shaped polisher makes an ideal instrument for this work. The medicinal properties of a freshly made solution of

| | | |
|---|---|---|
| Iodin crystals, | 10 gr. | |
| Potassium iodid, | 1 gr. | |
| Glycerin, | 4 dr. | M. |

to moisten the point on which the pumice is carried is very beneficial to the tissues in polishing these pockets, but this solution must be fresh, as it loses its penetrating properties when much over three days old. Of course all pumice must be rinsed from the pocket. If a pocket is deep enough or so shaped as to retain it, Beck's bismuth paste is a very good dressing. It keeps out all debris and is very healing to the tissues. It may be claimed that this polishing will break up healthy granulations, but it will not impede the the process of healing nearly as much as if the infectious matter is allowed to remain.

Any normally shaped tooth can be saved if the pocket has not extended beyond the apex, if root absorption has not commenced, and if at least one-third of the original attachment is intact. It is usually advisable to extract teeth that have much up-and-down motion, although a great many seemingly unsavable teeth can be saved by curing the disease and splinting properly. If one or two roots be entirely detached or unsavable they should be excised, and a good root or roots be utilized for crown or bridge work. Roots that cannot be cured should be removed.

### PROPHYLAXIS.

Prophylaxis, to be ideal, should start with the eruption of the deciduous teeth. In additional to an occasional visit of children to the dentist, mothers or nurses should be taught how to use a polisher and ribbon floss, and to watch for and remove all the foreign substances sure to gather on the teeth of most children. Children of six, seven, or eight years of age frequently become so interested in and educated to clean tooth surfaces that they detect foreign substances on their own teeth, and learn to handle a polisher as well as a brush with considerable ability. As the permanent teeth erupt, the fissures of molars and bicuspids should be covered with cement, and kept protected until maturity, or longer if necessary. A cotton roll is placed on each side of the tooth to be covered. The mother, nurse, or an assistant is requested to help hold these in place, then, after drying the tooth with compressed air or a chip-blower, it is wiped with alcohol and dried again. The cement is mixed to about the consistence used in crown and bridge work, and the occlusal surfaces are covered with the material, which is pressed to place with the thumb or any finger, well vaselined, thereby preventing the cement from sticking to the finger. The pressure applied should squeeze out every particle of cement except that which is held in the fissures. It will then interfere in no way with the interlocking of the cusps or the articulation, but will prevent caries in the fissures in which it sticks. The copper cements probably have more adhesive property than the lighter-hued ones, but are somewhat objectionable on account of their color. The deeper the fissure the more it is liable to caries, but the sooner the cement is applied, the longer it will last in the fissure. These fissure fillings may require renewing from every two to five years, but they will surely prevent caries of fissures, which is three-fourths of the battle in juvenile patients.

Patients should be subject to thorough prophylactic treatment by a skilled hand for from every one to three months, according to the care they are able to give their own teeth. By prophylaxis I mean the removal of all foreign substances and polishing by hand. At least two properly shaped points are necessary in all cases. One broad flat point between three and four millimeters in width at the end, for polishing, and preferably concave on one side and convex on the other, is used for the buccal and lingual surfaces of the teeth. A No. 12 gage shoe-peg, sharpened or made very thin to go as far as possible into the interproximal spaces, makes a very serviceable point for the second polishing. Then an iodin disclosing solution is applied, composed of—

| | | |
|---|---|---|
| Iodin crystals | 50 gr. | |
| Potassium iodid, | 15 gr. | |
| Zinc iodid, | 15 gr. | |
| Glycerin, | 4 dr. | |
| Water, | 4 dr. | M. |

Put up in a glass-stoppered bottle.

*Sig.*—Disclosing solution.

The teeth are stained with this solution and polished until it will disclose no more foreign substances. A dental engine polisher does the hardest polishing where it is least needed, and every time a revolving rubber cup or brush wheel touches the gum it cuts, making a scar; cicatricial tissue contracts. In other words, recession of the gums is frequently caused by careless dental engine polishing as well as by improper handling of a tooth-brush.

By ideal prophylaxis treatment we should be able to bring a child to manhood or womanhood with a comparatively sound set of teeth, very small fillings, if any, and well educated as to how a clean set of teeth feels.

### INSTRUCTIONS TO PATIENTS.

When a new patient presents himself, if he be not suffering pain, he should be instructed as to the presence of deposits on his teeth. This can be done by painting the surfaces of the teeth with the disclosing solution. This will not stain the tooth surfaces at all, but only the debris lodged thereon. The patient is given a hand mirror and shown how much accumulation of deposits there is on even apparently clean teeth. It is suggested to him that if as much infectious and decaying matter were allowed to remain on one portion of his hand for weeks and weeks as is allowed to remain on the teeth, he would be very much surprised if the tissue surrounding such a mass did not become inflamed and hypersensitive, as is often the case with the tissues surrounding the teeth. While the deposits are being removed, and the rough surfaces planed or filled and polished smoothly, it is explained to the patient that the enamel of teeth consists of from 95 to 98 per cent. calcium salts (See Tomes' "Dental Anatomy," page 14); that caries is merely a chemical reaction between an alkali—the enamel—and acids—the result of fermentation—just as much as the rusting of a piece of steel is a chemical reaction, and is just as preventable by polishing; that starches are converted into sugars and sugars into acids, and that all damage takes place under plaques—scales—*and only under plaques*, that free acid in the saliva does not destroy the enamel, but that no mouthwash can reach the acids under these plaques, and that no amount of brushing seems to entirely remove them, but that frequently improper brushing does a great deal of injury to the gums and tooth surfaces. The patient should also be instructed in the proper way of handling a brush. The essayist's plan is always to begin brushing on the grinding surfaces of the posterior teeth with a backward and-forward motion, keeping the bristles pointed in the direction of the roots, rotating the brush from side to side so that the bristles are felt on the gums on both the lingual and buccal surfaces; by this procedure the fissures are cleaned. Then the brush is placed well up on the gums and with a rolling motion moved toward the occlusal surface, *i. e.* up on the lower and down on the upper teeth, on both the lingual and buccal surfaces. When brushing the lingual surfaces of lower molars, the tongue should be drawn well back so as to expose those surfaces of the teeth to the brush. I usually recommend rather small brushes of medium texture. The smaller adult Rolling brush, medium, will meet the requirements in the majority of cases, and a much smaller brush should be used on lingual surfaces of anterior teeth—for a large one bridges over the inside of the arch. Enough brushes should be kept on hand all the time, so that each brush is used only once in twenty-four hours, and the teeth and gums should be brushed after each meal. Still, the tooth-brush, at best, is a very poor instrument with which to get the surfaces of the teeth cleaned, and if improperly handled does more harm than good. Cutter's ribbon floss should be used to polish the approximal surfaces once each day. Care should be taken when passing this floss between the contact points, not to allow it to snap into the gum tissue.

This procedure, with periodical visits to the dentist, will keep the teeth esthetically clean, but we want more (the staining solution will show how much more)—namely, prevention of caries. For five or six years I have been giving my patients orange-wood sticks and suitably shaped instruments of the type of porte-polishers to carry wooden points with which to polish off the debris which the tooth-brush leaves, and 75 per cent. of my patients have become quite proficient in the use of these accessories. Some patients are very awkward when they first try to use a polisher, but what child is not awkward in his first attempt to use a tooth-brush? It is only by repeated and persistent instructions and demonstrations that the majority of patients arrive at any degree of proficiency with either a brush or a polisher. Any person is awkward the first time he tries to use a pen, but persistent practice makes perfect. I frequently use this illustration in instructing patients.

Patients are provided with the formula for the disclosing solution, a mouth-mirror a polisher, and a small bottle of Buffalo flour of pumice. After a few months, as all rough places are polished down to a shining surface, I give them XXX silex, which is amorphous and therefore even less wearing than the crystals of pumice, and later I recommended a tooth-powder composed of—

| | |
|---|---|
| Precipitated chalk, | 6 oz. |
| Flour of sulfur, | 1 oz |
| Oil gaultheria or cassia | q.s. to flavor. M. |

*Sig.*—Tooth-powder.

Even rubbing with the wood points alone takes off debris that the brush failes to remove. Sodium bicarbonate used on the polisher is also very effective after the tooth surfaces have been made smooth.

The argument may be raised that patients will do damage in this way. A freshly extracted tooth can be cut considerably with a brush and precipitated chalk in thirty minutes of vigorous cross brushing, whereas three hours of rubbing with the same material on a wooden point makes no impression except to produce a beautifully polished surface. The only explanation I can give for this phenomenon is that the bristles of a brush are so hard that the whole crystal of grit embeds itself into the tooth, whereas the wood is so soft that the grit embeds itself in the wood, leaving just enough of it exposed to produce a polish.

In a few instances where patients have been abroad or for various causes knew they would be unable to present for regular prophylaxis treatment, in addition to the polisher, tape, etc., I have given them a scaler or two, and upon their return after six months or a year have been surprised to find their mouths in good condition, with practically no deposits, either soft or calcic, although previous to this *regime* of cleanliness deposits had gathered very rapidly. I mention this to show how thorough an intelligent person can be when given proper instructions.

By this care in quite a number of patients whose mouths were hypersensitive and whose teeth were decaying rapidly when this *regime* of clean surfaces was commenced, no new cavity has developed in six or seven years; their mouths are healthy, not one sensitive place in them, and of course, if care enough is taken to prevent caries, pyorrhea is an unknown quantity.

### PROPHYLAXIS DURING PREGNANCY.

There is no time when prophylaxis is indicated more especially than in pregnancy. The bodily resistance of the prospective mother is greatly reduced, and unless proper care is applied, caries progresses very rapidly. The salts which should be thrown out to protect the teeth go to make up bone and tissue for the coming child. The attending physician should recommend a visit to the dentist for the purpose of prophylaxis as soon as the patient's condition has been diagnosed. There should be a prophylaxis treatment every month. If the patient is not able to come to the dentist's office, she should have professional prophylaxis treatment at home, and as soon as she is able to go out, one of her first visits should be made to the dentist. Prophylaxis should be kept up once a month until the child is weaned and the mother returns to her normal healthy condition. The old saying of "A tooth for a child" should be proved a fallacy. Whenever I have been able to control these patients they have passed this trying period without even a decalcified spot forming on any of their teeth.

Unfortunately we cannot always practice ideal prophylaxis but are obliged to take cases as they are presented to us. If the patient is not suffering pain, the first measure is to clean the teeth. This not only arrests the process of destruction and puts the mouth in a condition more pleasant for treatment, but it allows us to make suggestions which should be very beneficial to the patient. If it is possible to get only one, two or three teeth clean in the first hour's sitting, this should be done well; one surface is scaled, smoothed, and polished until it is finished, before work is started in another place. The patient is taught how to use the iodin disclosing solution, a polisher and ribbon floss, and it is impressed upon him that at the next sitting the disclosing solution should leave no stain in any portion of the teeth that have been gone over at the present sitting; that at the next sitting as many more teeth will be cleaned as possible, and so on until the entire mouth is put in a healthy condition. Painting the teeth with the iodin disclosing solution once or twice a week will, by virtue of the disinfecting property of the iodin, prevent a great deal of caries and tend to keep the gums hard and healthy, but its principal value lies in locating plaques which are transparent, hence invisible to the naked eye.

Shallow, cervical cavities can be ground, filed, or planed out, and a high polish imparted to the tooth at this point. This may leave some sensitive places, but by using sodium bicarbonate the tenderness can be relieved so that they can be kept clean. Milk of magnesia may be recommended for its acid-neutralizing properties. Painting with delinquescent zinc chlorid does a great deal toward relieving tenderness. I also frequently employ 10 per cent. silver nitrate. This does not stain any except actual carious portions, and greatly retards the progress of destruction. Patients should polish such places every day, as long as they are sensitive, with sodium bicarbonate. Tenderness usually disappears within from one to four weeks.

### FEES.

This work should command as high or even a higher fee per hour than we would receive if we were making fillings, inlays, crowns, bridges, or plate work, for we are rendering our patients better service, and a just compensation should be obtained.

### MISCELLANEOUS SUGGESTIONS.

All cavities should next be filled. If improperly contoured fillings be present, they should be replaced with properly contoured ones that are carefully finished at the cervical margins. When putting in fillings, the margins should be extended to where they are protected by gum tissue or can be reached easily with the brush, polisher, or ribbon floss, according to the principle of extension for prevention, thereby rendering the cavity margins as well as all surfaces of the teeth immune to caries by the process of absolute cleanliness. In other words, extension for prevention should be practiced, meaning the extension of the immune surfaces.

If bridge work which irritates the tissues, cannot be kept clean, or is improperly occluded, it should be replaced by appliances that meet the ideal requirements. It is impossible to maintain a healthy condition when a partial plate is worn which rests on the gums.

During the first year of prophylaxis treatment the patient should be seen at least once a month. After that, as he learns what a clean, healthy mouth feels like and how it is maintained, the periods for treatments may be extended, but it is not well to let any mouth go unobserved over three months.

One of my patients informed me that she was limiting herself to two meals a day, because it was too much work to use a polisher more than twice daily. I told her that if she went over her mouth twice a week it would be sufficient. She replied, "Oh, but my mouth feels so uncomfortable until I have used the polisher and ribbon floss." Needless to say she has not had a new cavity in several years, and barring accident or sickness, she will keep her teeth until she reaches the allotted threescore and ten.

Every dental school should have a chair of pyorrhea and prophylaxis. The head of this department should be chosen as carefully as the professor of operative dentistry. These two departments should be on the best of terms and work in harmony, *i. e.* fillings, crowns, bridges, etc., should be constructed so that they can be kept clean.

Students who have entered a dental college should have a careful examination made of their oral condition, records should be kept, and if these students are not giving their own mouths proper care, instructions should be given them. Examinations should be held periodically and their grading should partly depend upon this examination. In this way every graduate would know from experience the sensation of a clean, healthy mouth, for unless a man really knows and believes what he is advocating, his arguments lack force when he goes into practice and tries to instruct his patients.

Any student who lacks the ability or does not take pride enough in his own mouth or his chosen profession to practice preventive dentistry on himself should not be graduated.

By these measures we shall render to our patients better service in preventing caries and pyorrhea than by inserting the best filling, crown, bridge, or plate that human hands ever made.—*Cosmos.*

